# Extracellular Traps in Inflammation: Pathways and Therapeutic Targets

**DOI:** 10.3390/life15040627

**Published:** 2025-04-08

**Authors:** Stelvio Tonello, Nicole Vercellino, Davide D’Onghia, Alessia Fracchia, Giulia Caria, Daniele Sola, Paolo Amedeo Tillio, Pier Paolo Sainaghi, Donato Colangelo

**Affiliations:** 1 Dipartimento di Medicina Traslazionale, Università del Piemonte Orientale, Via Solaroli 17, 28100 Novara, Italy; nicole.vercellino@uniupo.it (N.V.); 20033432@studenti.uniupo.it (A.F.); 20033138@studenti.uniupo.it (G.C.); pierpaolo.sainaghi@med.uniupo.it (P.P.S.); 2 Dipartimento per lo Sviluppo Sostenibile e la Transizione Ecologica, Università del Piemonte Orientale, Piazza S. Eusebio 5, 13100 Vercelli, Italy; 3 Laboratory of Metabolic Research, IRCCS Istituto Auxologico Italiano, 28824 Oggebbio, Italy; d.sola@auxologico.it; 4 Clinical Chemistry Laboratory, Maggiore della Carità Hospital, 28100 Novara, Italy; paoloamedeo.tillio@maggioreosp.novara.it; 5 Dipartimento di Scienze della Salute, Farmacologia, Scuola di Medicina, Università del Piemonte Orientale, Via Solaroli 17, 28100 Novara, Italy; donato.colangelo@med.uniupo.it

**Keywords:** ETs, ETosis, NETs, NETosis, neutrophils, inflammation, immunity, strategies, therapies, pharmacological approach

## Abstract

New roles for immune cells, overcoming the classical cytotoxic response, have been highlighted by growing evidence. The immune cells, such as neutrophils, monocytes/macrophages, and eosinophils, are versatile cells involved in the release of web-like DNA structures called extracellular traps (ETs) which represent a relevant mechanism by which these cells prevent microbes’ dissemination. In this process, many enzymes, such as elastase, myeloperoxidase (MPO), and microbicidal nuclear and granule proteins, which contribute to the clearance of entrapped microorganisms after DNA binding, are involved. However, an overproduction and release of ETs can cause unwanted and dangerous effects in the host, resulting in several pathological manifestations, among which are chronic inflammatory disorders, autoimmune diseases, cancer, and diabetes. In this review, we discuss the release mechanisms and the double-edged sword role of ETs both in physiological and in pathological contexts. In addition, we evaluated some possible strategies to target ETs aimed at either preventing their formation or degrading existing ones.

## 1. Introduction

### 1.1. Context and Objectives

New roles for immune cells that go further than the classical cytotoxic response have been highlighted by growing evidence. Net-like DNA structures called extracellular traps (ETs) offer an interesting opportunity for the better comprehension of inflammatory, infective, and autoimmune diseases. Possible insights are also coming from the analysis of ETs’ involvements in special populations of patients. We reviewed some important data offered by the literature.

### 1.2. From Immune Cells to ET

In recent years, growing evidence has highlighted new roles for immune cells like white blood cells (granulocytes and macrophages), overcoming the classical cytotoxic response. These cells can undergo a different type of response based on the release of cellular DNA to form extracellular traps (ETs) [1,2,3].

Neutrophils, also known as neutrophilic granulocytes or polymorphonuclear neutrophils (PMNs), have historically been described in an acute inflammatory response, and they were considered short-lived antibacterial effector cells [4]. They play a crucial role in the immune defence against bacterial and fungal pathogens and participate in developing inflammatory reactions [5]. However, recent evidence suggests neutrophils are versatile and can perform previously unsuspected functions, including reverse transmigration and modulation of other innate and adaptive immune leukocytes, establishing an important cellular crosstalk [6]. Neutrophils are the most abundant white blood cells in human circulation, representing 50 to 70% of all circulating leukocytes [7]. Moreover, the circulatory half-life of human neutrophils is still a matter of debate; it is now estimated to be longer, ranging from 7 to 9 h up to 3.75 days [8]. Through the release of α-defensins, reactive oxygen species (ROS), elastase, cathelicidin, matrix metalloproteinases, cathepsin G, and ETs are involved in the antimicrobial and cytotoxic mechanisms of action against the non-self. Neutrophils show a significant biosynthetic capacity for C-C and C-X-C chemokines, which represent factors with immunoregulatory, proinflammatory, and anti-inflammatory roles as well as fibrogenic and angiogenic cytokines [6,9].

Evidence highlighted that neutrophils play a crucial role in acute infections and inflammation but are also implicated in chronic inflammatory disorders or aging-related diseases, such as atherosclerosis, psoriasis, rheumatoid arthritis, inflammatory bowel disease, diabetes, and cancer [10]. Excessive activation of these cells may be responsible for the development of multiple organ dysfunction syndrome. In this context, the lungs represent the main target of this condition, the so-called acute lung injury (ALI) and its more severe form is the acute respiratory distress syndrome (ARDS) [5,10]. Although neutrophils are mostly considered beneficial to the host, their improper activation may also lead to tissue damage in the context of an autoimmune or exaggerated inflammatory reaction [11,12].

Among granulocytes, there is another class of cells that can produce ETs, eosinophils, a cell family whose classical mechanism of action is based on the presence in their cytoplasm of acidic granules released after pathogenic stimulation. As observed in neutrophils, eosinophils, after appropriate stimulation, could undergo decondensation and release DNA in the extracellular milieu. The only difference between these processes is the timing of ETs production and the fate of the specific cytoplasmic granules. ET fibers are generated more rapidly in eosinophils than in neutrophils, and acidic granules remain intact and associated with histone proteins in the final ET structure [13,14,15,16].

This type of response mechanism is not restricted to granulocytes; indeed, it has also been observed in monocytes/macrophages [3]. It is well known that circulating monocytes are extravasated after pathological stimulation and become active macrophages. Physiologically, these cells remove the noxious stimuli, as well as cellular debris, by phagocytosis. In some conditions, they have been described as being able to start releasing DNA fibers originating from the so-called Monocyte/macrophage extracellular traps (METs) to entrap the non-self-material that would be subsequently phagocytosed [3].

### 1.3. ETs Production

In 1996, it was discovered that neutrophils, after chemical stimulation with Phorbol Myristic Acid (PMA), could undergo a death pathway different from apoptosis and necrosis [17]. A typical aspect of this new death modality is the disruption of the nuclear and granular membranes. This kind of neutrophil suicide is characterized by the release of web-like structures formed by chromatin and antimicrobial factors, called neutrophil extracellular traps (NETs) [18,19]. The mechanism through which NETs are produced is called NETosis, and it contributes to the risk of thrombosis in inflammatory disorders, activating the coagulation process. The evidence suggests the discovery of two mechanisms of NETosis: vital NETosis, in which fiber expulsion occurs without altering the membrane of neutrophils, and suicidal NETosis, where PMNs die after removing the filaments [20].

**Figure 1:**
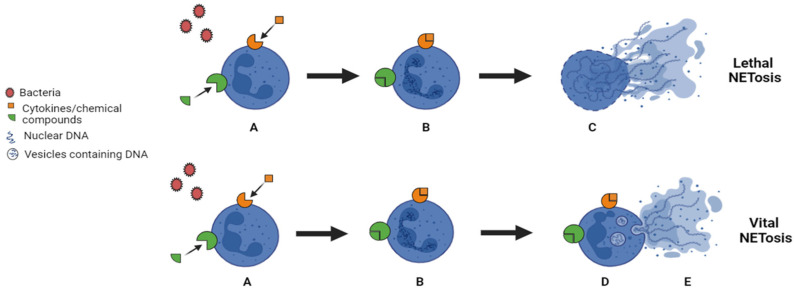
ETosis process in PMNs: lethal and vital NETosis. (**A**) Different stimuli activate neutrophils: cytokines, chemical compounds, and bacteria. (**B**) Activated white blood cells react by changing the structure of the nuclear membrane and start to lose their integrity. (**C**) Release of NETs in the extracellular space with the consequent cell death (Lethal NETosis). (**D**) Formation of vesicles containing DNA after the rupture of the nuclear membrane. (**E**) Migration of vesicles with entrapped DNA to the cell membrane and NETs release, leaving the cell alive (vital NETosis). Image created with BioRender 2025.

In 2007, Nicotinamide Adenine Dinucleotide Phosphate (NADPH) was discovered to be crucial in disrupting the membranes and mixing nucleic acid and granular protein [19]. As stated before, the mechanism of ETs production is similar in all cell types, and it is called ETosis. The release of ETs occurs after membrane perforation and cell lysis, which are used to entrap particulate matter such as bacteria. Thus, the following evidence highlights that not only PMA but also IL-8, bacteria, fungi, protozoa, antibody–antigen complexes, autoantibodies [21], TNF, INF [22], and further stimuli trigger ETosis [23], as shown in Figure 1. ETosis could be considered a form of beneficial cellular suicide [12]. Furthermore, its mechanism of action involves the activation of different cellular receptors, such as Toll-like receptors (TLRs). Specific receptor stimulation triggers the activation of several intracellular signaling pathways, many of which involve ROS generation, NF-kB activation, and autophagy [23].

### 1.4. The Molecular Mechanism Involved in the ETosis Process

**Figure 2:**
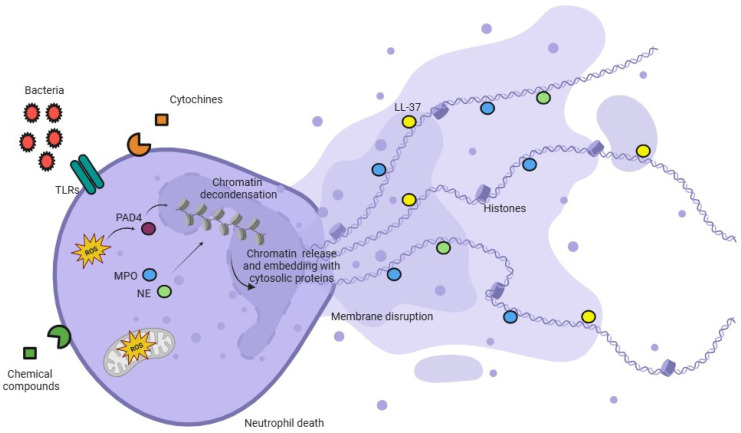
Molecular events of lethal NETosis. TLRs = Toll-like receptor; PAD4 = peptidyl arginine deiminase 4; LL-37 = human antimicrobial peptide; MPO = myeloperoxidase; NE = neutrophil elastase. Image created with BioRender 2025.

NETosis could be considered a model to describe ETs formation in all the immune cells studied. Loss of chromatin segregation into heterochromatin and euchromatin represents another hallmark of NETosis and of other types of ETs production [24]. In this process, many enzymes, such as the ones of azurophilic granules, elastase, and myeloperoxidase (MPO), are involved; the latter can move into the nucleus in the earliest stage of ETosis in a particular way. Elastase is the first enzyme that reaches the nucleus, and, by cleaving the H1 linker, modifies the histones’ core [24,25]. Elastase is also crucial in trap formation; indeed, it has been observed that mice deficient in this enzyme cannot produce ETs [24]. Whilst MPO enters the nucleus later, its activity is correlated with the enhancement of chromatin decondensation caused by hypochlorite acid synthesis [24,26]. Another enzyme that enters this suicidal pathway is Peptidylarginine deiminase 4 (PAD4). PAD4, after proper activation, induces deamination of the arginine residues to citrulline in histones 3 and 4, leading to a weaker DNA binding; in the literature, it has been shown that histones, as well as decondensed chromatin, are citrullinated [26,27,28,29]. Figure 2 resembles the molecular mechanism of NETs release.

The role of PAD4 in ETosis was studied in knock-out mice, demonstrating that these mice could not produce ETs [25,30,31]. Recently, it has been demonstrated that autophagy, which is essential for many immune functions, such as degranulation and ROS production, is also involved in ETs production, and this phenomenon is NADPH oxidase-dependent [32,33]. PMA stimulation of white blood cells resulted in giant vacuoles, the autophagosomes. Even if the general mechanism of this process is known, the underlying molecular mechanism is not fully understood, and it has been hypothesized that mTOR and PTEN could play a key role [34].

Neeli et al. demonstrated that the cytoskeleton regulates ETosis, with tubulin crucial in directing the granules’ exocytosis and phagocytosis [32,35,36].

Furthermore, recent in vivo studies have also demonstrated that the ETs production mechanisms in granulocytes could undergo specific pathways during aging, involving Atg5 and TLR2 [37]; while drug therapy and siRNA could inhibit such a phenomenon directed against mTOR and LC3 [38].

Despite the large body of evidence describing autophagy involvement in NETosis, the above-illustrated results are not universally accepted: Germic and coworkers have shown that ATg5-knock-out mice can retain NET-producing ability while showing reduced autophagy [39]. Moreover, it has been observed that PI3K inhibitors, such as chloroquine and A1 bafilomycin, block late autophagy but do not interfere with NET production, suggesting the possibility of an independent autophagy pathway being followed in NET formation [16,40,41,42,43,44,45].

### 1.5. ETs and Pathology: A General Overview

There are growing pieces of evidence suggesting ETs, particularly NETs, are involved in the onset and progression of several pathologies. As inflammation is universally recognized as a key aspect of many pathological states, neutrophil recruitment in such situations is unsurprising. In recent years, many clinical studies have focused on NETs’ correlation with autoimmune diseases (e.g., psoriasis), chronic inflammatory diseases, and neurodegenerative diseases. Moreover, there is a large body of evidence correlating ETs to cancer onset and progression, but this is out of the scope of the present review [6,27,46,47,48,49].

### 1.6. ETs and Skin Affections

Psoriasis is a common, chronic, and autoimmune dermatological condition characterized by an excessive inflammatory response directed against self-tissues. This pathology has a high incidence, affecting approximately 2–3% of the world population, and geographical location, age, ethnicity, sex, genetic, and environmental factors are strictly associated with the development of this condition [50]. It has been demonstrated that in some skin inflammatory diseases, such as acne, the roles of NETosis and inflammosomes are very important since they are closely related to the innate immune response. Inflammosomes act as one of the first factors in immune responses. The process that leads to the secretion of inflammatory cytokines interleukin 1β (IL-1β) and interleukin 18 (IL-18) is one of the mechanisms of response to external agents, such as bacterial infections, where NETosis is also involved. The activation of proteins present in the cytosol of cells, like the nucleotide-binding domain, leucine-rich family, and pyrin domain-containing-3 (NLRP3), leads to the inflammasome, one important factor involved in NETosis. Some authors have suggested that the inhibition of NETosis might represent possible treatments for inflammasome-mediated inflammatory diseases [51].

Psoriasis can occur at any age, particularly common in two age groups of 16–22 and 55–60 years [52]. This pathology is also associated with a dramatic reduction in patients’ quality of life, since it also has strong psychological implications. From a clinical point of view, skin symptoms are associated with many other comorbidity syndromes, such as psoriatic arthritis and cardiovascular diseases [53].

From a molecular point of view, the primary inflammatory cytokine involved in psoriasis development is IL-17, an inflammatory mediator that has been proven to induce ETs production in all immune cells considered [54,55]. In particular, the specific release of NETs happens as a result of the activation of the infiltrated neutrophils in the epidermis, a typical hallmark of psoriasis [56]. A variety of mechanisms, such as the stimulation of the Toll-Like Receptor 7 and 9 (TLR7 and TLR9) by LL37-DNA [57], the inflammasome activation [58], the macrophage pyroptosis [59], and the activation of IL-36 cytokines are enhanced by the neutrophils and NETs production. Among them, it has been observed that, in vitro and using a common mouse model of psoriasis-like inflammation [60], Toll-like Receptor 4 (TLR4) signaling and the activation of IL-36 by neutrophils are able to enhance skin inflammation through NETs release. These two players, acting in a synergistic manner, stimulate LCN2 expression, a neutrophil chemoattractant, in keratinocytes, resulting in increased neutrophil infiltration and inflammation of the skin [56].

Clinical evidence supports the hypothesis that psoriasis progression is associated with an increased ETs production due to the autoimmune response [54,55].

Even if it is well known that prominent levels of circulating ETs are dangerous, some recent in vitro experimental evidence suggests that low NETs doses could positively affect normal skin regeneration as they stimulate keratinocytes proliferation through NF-kB signaling pathway activation [10]. Such new experimental evidence declares that a physiological balance in ETs presence could exist, thus fostering the possible development of innovative personalized therapeutic medicine approaches that are useful when a rapid reconstitution of the skin integrity is needed.

### 1.7. ETs and Chronic Inflammatory States in Smokers

Smoking is a diffuse social habit associated with many side effects, ranging from inflammation to lung cancer, emphysema, and atherosclerosis. Nicotine-noxious effects could be observed in the oropharyngeal, respiratory, and circulatory cavities. Nicotine-induced inflammation is associated with the induction of neutrophils’ degranulation and the inhibition of their phagocytic activity. Another well-known side effect of nicotine exposure is the increase in ROS generation, a physiological condition correlated to ET production [36,61,62,63,64,65,66].

A common condition mainly associated with cigarette smoke is chronic obstructive pulmonary disease (COPD), which represents a relevant public health issue with high morbidity and mortality globally [67]. Moreover, it has recently been observed that COPD severity is linked to the overproduction of NETs in the airways [68] and also airway microbiota diversity and COPD exacerbation frequency [69]. As in other conditions, NET release can appear as a double-edged sword [70]; indeed, on one hand, it represents a strong antimicrobial strategy, but on the other hand, it plays a proinflammatory and cytotoxic role, resulting in autoimmunity and tissue damage [28]. In particular, in COPD, airflow limitation and airway inflammation correlate with NETs and extracellular DNA levels in sputum [71].

### 1.8. ETs and Lung Pathologies

Asthma is a respiratory disease characterized by a reduced air supply to the lungs due to airway wall remodelling associated with chronic inflammation and bronchospasm. Some in vivo studies have shown a direct correlation between asthma development and ETs production. A similar correlation has also been observed in humans, where ET production in asthmatic patients was associated with IL-8, IL-17, and ROS production, all factors that are known to increase ET generation and release, thus worsening the clinical setting [66,72,73]. Moreover, it has been reported that double-stranded DNA (ds-DNA), myeloperoxidase-DNA (MPO-DNA), and citrullinated histone H3 (CitH3), which are putative surrogate biomarkers, have been detected in several sites and fluids in asthmatic patients [74]. Recently, Granger et al. discovered that both circulating NETs and eosinophil extracellular traps (EETs) represent novel biomarkers for severe asthma (SA) [75]. SA condition affects 10% of the asthmatic patients, where even in the presence of stable high-dose inhaled corticosteroids (ICSs), asthma exacerbations occur more frequently [76]. In the literature, it has been reported that a higher level of EET-positive eosinophils is present in the peripheral blood of SA patients compared to non-SA patients and healthy subjects [77]. Furthermore, in asthma, altered mitochondrial function is observed: mitochondrial biogenesis and respiration are increased resulting in abnormal cellular phenotype in airway smooth muscle cells and epithelial cells [78,79,80]. Therefore, the role of mitochondria is crucial in eosinophil function and survival and EET formation [81]. EETs, released by live cells, are made of granule proteins such as the eosinophil cationic protein (ECP), major basic protein (MBP), and DNA, which has a mitochondrial origin without the presence of the expression of mitochondrial DNA genes and histone proteins [82].

Another lung disease associated with ET production is cystic fibrosis (CF), an autosomal recessive inherited disease characterized by an altered expression and activity of the CF transmembrane regulator (CFTR) gene and protein, leading to an abnormal accumulation of a thick mucus layer that could be in favour of the colonization and growth of exogenous bacteria. Moreover, the loss of function of the CFTR gene is in part involved in the defective function of the macrophages [83,84]. Some authors have described that prolonged hypoxia might affect the development of interstitial pulmonary fibrosis in COVID-19 patients. In fact, hypoxia, together with fibroblast duplication and the deposition of extracellular matrix (ECM), modulates hypoxia-inducible factor 1-alpha, (HIF-1-alpha) functions at multiple levels of the fibrotic process [85,86]. It has been shown that several mechanisms contribute to the release of macrophage extracellular traps (METs), such as augmentation in intracellular calcium, oxidative stress, IFN-γ, TNFα, extracellular DNA, drug exposure (Fosfomycin, statins), and interaction with microbes (bacteria, mycobacteria, yeast) [87]. Recently, some authors attributed to exogenous neutrophils elastase (NE) a crucial role in METs production [88]; indeed, they observed that the internalization and the activation of the proteolytic function regarding NE in the macrophages result in H3 citrullination and “histone H3 clipping”, events which anticipate the METs production [88]. These particles, such as NETs and ETs, can promote inflammation in the airway’s microenvironment and in the lung parenchyma, where histones, high-mobility group box 1 (HMGB-1), myeloperoxidase, extracellular DNA, and proteinases play a crucial proinflammatory role [89]. However, METs represent the main source of ET in CF and the previously mentioned COPD patients [88].

Accordingly, clinical evidence has emphasized the correlation between ET levels in accumulating mucus and bacterial infection; indeed, DNA fibers can act as a scaffold, favouring microorganisms’ growth. Considering these observations, ET levels evaluation could represent a possible way to evaluate pneumonia severity [90,91,92].

### 1.9. ETs and Kidney Disease

Some clinical works have also demonstrated ET’s involvement in the development and progression of renal diseases. These authors correlated ETs production to acute kidney damage caused by the chronic inflammation of histones released from ETs. Defective NETs clearance is also associated with the production of autoantibodies against NETs themselves: antinuclear antibodies and antineutrophil cytoplasmic antibodies (ANCAs) [93], which can lead to pauci-immune glomerulonephritis [94]. Moreover, it has been hypothesized that the ETs’ involvement in the necro-inflammatory early stage of acute tubular necrosis observed in renal vasculitis, anti-glomerular basement membrane disease, lupus nephritis, and thrombotic microangiopathies [95,96] results in local and collateral damage. Gupta et al. have shown that NETs and their biomarkers are also involved in diabetic kidney disease (DKD) [97], one of the most crucial complications of diabetes mellitus [98] and its severity. They demonstrated that, in vitro, in diabetic mice and humans, NETs are able to contribute to the stimulation of NLRP3 inflammasome and IL-1β signaling in glomerular endothelial cells, resulting in glomerular endothelial dysfunction due to high glucose levels. Through PAD4 inhibition, low amounts of glucose-induced NETs are released, avoiding inflammation, renal damage, and endothelial dysfunction [97]. The production of NETs, indeed, can interfere with and decrease eNOS phosphorylation and the integrity of the endothelial barrier increasing levels of soluble VCAM-1 into the plasma, leading to endothelial dysfunction. Endothelial dysfunction, as indicated by albuminuria, reflects systemic endothelial injury and an increased risk for vascular diseases. These findings underline the significant importance of NETs formation and activity also in cardiovascular and metabolic diseases such as atherosclerosis, myocardial infarction, diabetes, and obesity [99,100,101,102,103].

### 1.10. ETs and Neurodegenerative Disease

Neutrophils play a significant role in the development of many neurodegenerative diseases, such as Alzheimer’s disease (A.D.). A.D. is a neurodegenerative disorder leading to a progressive deterioration of cognitive functions. It is characterized, at the cellular and tissue level, by β-amyloid accumulation in the neural parenchyma, causing inflammation and subsequent neuron death [104]. Many studies have highlighted neutrophil accumulation in cerebral blood vessels and their ability to release DNA fibers upon inflammatory stimulation; in the literature, it has been reported that the accumulation of ETs commonly occurs in the cerebral vessels of people with A.D. [95,104,105]. In neurons, the gathering of β-amyloid contributes to the production and activation of the factors of the complement system, such as C1q, CR1, and C5a, which are involved in the assembled neutrophils and NET release into the brain [106]. In addition, it has been reported that the complement system is not able to clean NETs, resulting in NET accumulation and overstimulation of the inflammatory complement system cascade, leading to neuronal damage [106,107].

A further study underlined the presence of a higher amount of MPO+ cells releasing NETs both inside blood vessels, in the cortex and hippocampus of individuals with A.D., compared to age-matched controls. Moreover, these authors found that in 5xFAD and 3xTg-AD mice, two transgenic mouse models of A.D., LFA-1 integrin mediates cognitive impairment and pathogenesis of A.D. through NETs formation [108].

Such clinical evidence suggests that ETs accumulation could play a pivotal role in exacerbating neuroinflammation and suggests the possibility of targeting them with innovative therapeutic approaches to treat such disease [95,104,105].

### 1.11. ETs and Liver Disease

Some studies have correlated ETs production and release also to liver diseases such as non-alcoholic steatohepatitis (NASH) and hepatocellular carcinoma (HCC), induced by different stimuli, as well as hepatitis B virus [109,110,111].

In NAFLD conditions, the lipid accumulation in the liver can lead to inflammation with neutrophils as active players which are able to release NETs. The latter overstimulates the inflammation status and retrieves several immune cells in the liver, such as T regulatory cells (Treg) and macrophages, leading to the progression from NASH to HCC [112,113]. Indeed, it has been reported that cancer progression and metastasis involvement can be enhanced by NET formation [114]. HCC cells are trapped by NETs, which promote the stimulation of the TLR4/9-COX2 axis, involved in the inflammatory response and metastatic process [115].

Moreover, it has been observed that the NET level is higher in HBV-positive HCC and is implicated in HBV-related hepatocarcinogenesis, creating an inflammatory tumor microenvironment (TME); indeed, circulatory NETs represent a tool to predict extrahepatic metastasis of HBV-related HCC. A huge NETs production is the consequence of the stimulation of TLR4/RAGE-ROS molecular pathway mediated by HBV-induced S100 calcium-binding protein A9 (S100A9), involved in HCC cells’ growth and metastasis [116].

Both in vivo experiments and clinical observational studies have demonstrated a strong correlation between circulating free DNA fibers and the development of these pathologies, suggesting their use as a potential clinical biomarker for diagnostic purposes. ET’s interest in the hepatological clinical field is still growing, and some in vivo studies have shown that a fatty acid-rich diet can increase ETs production [117,118,119,120]. In contrast, recently, it has been shown that in obese mice fed with a high-fat diet (HFD), neutrophils produce a reduced amount of NETs compared to lean controls under a normal diet (ND). According to the results, it has been found that ND neutrophils in both physiological and inflammatory conditions produce NETs through glycolysis and/or the pentose phosphate pathway. On the other hand, these strategies are involved in NETs release in cells of septic HFD mice, while they show an “exhausted phenotype” after a secondary ex vivo activation, resulting in reduced NETs production despite high glycolytic potential and flexibility to oxidize fatty acids [121].

## 2. Therapeutic Strategies

As reported in Table 1, the production and release of NETs can be modulated by several mechanisms of action of different molecules. Raf-MEK-ERK signaling, phosphorylation of the p65 subunit of NF-κβ via Rac2, PAD4, calcineurin signaling, blocking receptors with monoclonal antibodies, and DNAases represent some of the molecular pathways involved in decreased NET formation [122].

## 3. Conclusions and Future Perspectives

NETs have a controversial role. On the one hand, NETs are an efficient system through which the innate immunity can protect the host and prevent microbes from spreading; on the other hand, defective overproduction and accumulation of these free DNA fibers result in several pathological scenarios, such as cancers, infectious, non-infectious, and autoimmune diseases. Although novel therapeutic strategies targeting NETs are emerging, further and more extensive research is needed to explore their impact on different diseases.

While considerable progress has been made in understanding ETs’ biology, further research is essential to fully elucidate their specific molecular pathways in diverse diseases. Identifying disease-specific ET biomarkers could provide novel diagnostic and prognostic tools. Additionally, developing precise therapeutic strategies that balance ETs production and clearance is crucial to avoid unwanted side effects while preserving host defense. ET-targeted therapies promise precise and personalized medicine approaches in inflammatory and autoimmune disorders.

## Figures and Tables

**Table 1:** In the following table are reported the compounds described in the literature as a NET modulator and the published findings.

Authors	Year	Reference	Anti-NETs	Main Findings
Chen et al.	2023	[123]	Simvastatin	In a mouse model of severe asthma, Simvastatin decreases Th17-mediated neutrophilic inflammation and airway hyperreactivity through the reduction in PAD4 expression, resulting in NETosis inhibition.
Demkow U	2023	[124]	DNase I and II families 3’-exonucleases (TREX1 and TREX2)	These families of DNAse and exonucleases are involved in NETs degradation.
Shen et al.	2022	[125]	Cl-amidine	It has been shown that Cl-amidine is involved in the inhibition of PAD4-dependent NETs formation, avoiding NETosis. NET inhibition with Clamidine alleviated neuroinfammation, neuronal apoptosis, and neurological deficits through STING-dependent IRE1α/ASK1/JNK signaling pathway in mice with traumatic brain injury.
Shi et al.	2023	[126]		
Wang et al.	2023	[68]	GW31161A	GW311616A represents a selective and potent inhibitor of neutrophile elastase (NE). It prevents cigarette smoke extract (CSE)-induced NETs formation through the blocking of NE nuclear translocation and subsequent chromatin decondensation. In vivo, GW311616A reduces pulmonary generation of NETs, attenuates neutrophil numbers and percentages, and the levels of neutrophil chemotactic factors and proinflammatory cytokines in BALF. Moreover, it is able to improve lung function in the COPD mouse model.
Wang et al.	2023	[68]	CXCR2 antagonist	It blocks the trafficking of the neutrophil from the blood into the airways. Additionally, in COPD patients, the CXCR2 antagonist partially inhibits spontaneous NETosis in sputum neutrophils of COPD patients.
Zang et al.	2019	[127,128]	Erythromycin	Erythromycin inhibits CSE-induced NET formation. In mice chronically exposed to cigarette smoke, erythromycin decreases NETs in the airway and ameliorates emphysema by downregulating Th1 and Th17 cells and suppressing CD40+ and CD86+ mDCs.
Farrera et al.	2013	[128]	Cytochalasin Nystatin	The administration of cytochalasin, an inhibitor of actin polymerization, and nystatin, belonging to the endocytosis inhibitors family, are involved in NETs clearance mediated by the macrophages, allowing an active and endocytosis-dependent process.
Sollberger et al.	2018	[129]	Molecules based on the pyrazolo-oxazepine scaffold (LDC7559)	A class of molecules based on the pyrazolo-oxazepine scaffold is capable of inhibiting gasdermin D and consequently NET formation. LDC7559 is able to inhibit PMA-induced NET formation without interfering with phagocytosis and impacting the physiological activity of Nox, NE, or MPO.
Totani et al.	2021	[130]	Roflumilast	They are selective PDE4 inhibitors. Roflumilast N-oxide (RNO) blocks prothrombotic functions: it inhibits Pyk2 and Akt phosphorylation, and curbs NETs production by PMNs adherent on fibrinogen-coated surfaces, and it has been approved as an adjuvant to reduce the risk of exacerbation in patients with severe COPD. Apremilast, inhibiting PDE4, prevents the activation of neutrophil surface markers as well as NETosis, ROS production, intracellular signaling and genes, and pathways related to innate immunity, and chemotaxis. Rolipram strongly affects PMA-induced NETosis and inhibits isolated neutrophil adhesion.
Le Joncour et al.	2023	[131]	Apremilast	
Shishikura et al.	2016	[132]	Rolipram (PDE4 inhibitors)	
Yoshimoto et al.	2023	[133]	Dual antiplatelet therapy (DAPT): aspirin and ticagrelor	Suppressing NETs production and avoiding platelet activation may prevent the implantation of intrahepatic cholangiocarcinoma (iCCA) cells.

## Abbreviations

The following abbreviations are used in this manuscript:

NETs	Neutrophil extracellular traps
ET s	Extracellular traps
MPO	Myeloperoxidase
PMNs	Polymorphonuclear neutrophils
ROS	Reactive oxygen species
ALI	Acute lung injury
ARDS	Acute respiratory distress syndrome
METs	Monocyte/Macrophage extracellular traps
PMA	Phorbol Myristic Acid
NADPH	Nicotinamide Adenine Dinucleotide Phosphate
TLRs	Toll-like receptors
COPS	Chronic Obstructive Pulmonary Disease
Ds-DNA	Double-stranded DNA
MPO-DNA	Myeloperoxidase-DNA
Cit-H3	Citrullinated histone
EETs	Eosinophil extracellular traps
SA	Severe asthma
ICSs	Inhaled corticosteroids
ECP	Eosinophil cationic protein
MBP	Major basic protein
CF	Cystic fibrosis
CFTR	Cystic fibrosis transmembrane regulator
NE	Neutrophils elastase
HMGB-1	High-Mobility Group Box 1
ANCAs	Antineutrophil Cytoplasmatic Antibodies
DKD	Diabetic kidney disease
A.D.	Alzheimer’s disease
NASH	Non-Alcoholic Steatohepatitis
HCC	Hepatocellular carcinoma
TME	Tumor microenvironment
S100A9	S100 Calcium-Binding Protein A9
HFD	High-fat diet
ND	Normal diet
CSE	Cigarette smoke extract
RNO	Roflumilast N-Oxide
DAPT	Dual antiplatelet therapy
iCCA	Intrahepatic cholangiocarcinoma
